# The association between dietary inflammatory index and anemia in individuals with diabetes mellitus

**DOI:** 10.3389/fnut.2025.1538696

**Published:** 2025-02-17

**Authors:** Nengneng Cao, Jinlan Li, Chun Ling, Jiajia Wang, Furun An

**Affiliations:** ^1^Department of Hematology, The Second Affiliated Hospital of Anhui Medical University, Hefei, Anhui, China; ^2^Anhui Medical University, Hefei, Anhui, China; ^3^Department of Hematology, Affiliated Chuzhou Hospital of Anhui Medical University, Chuzhou, Anhui, China; ^4^Department of Hematology, Tongling People's Hospital, Tongling, Anhui, China

**Keywords:** dietary inflammatory index, anemia, cross-sectional study, National Health and Nutrition Examination Survey, diabetes mellitus

## Abstract

**Background:**

Diabetes mellitus (DM) has emerged as a significant component of the global health crisis, closely linked with anemia. Studies have demonstrated a connection between anemia and inflammation. The Dietary Inflammatory Index (DII) is a novel metric for evaluating the overall inflammatory or anti-inflammatory impact of dietary intake. While risk factors for DM are linked to an unhealthy diet, there is currently insufficient research regarding the correlation between DII and anemia among individuals with DM. This study aims to investigate the correlation between DII and anemia among American individuals with DM.

**Methods:**

This study utilized data from the National Health and Nutrition Examination Survey (NHANES) database, encompassing 4,435 participants recorded between 2007 and 2016. We conducted a weighted multivariable logistic regression analysis to investigate the correlation between the DII and anemia of individuals with DM. Furthermore, we explored their relationship through subgroup analysis and smooth curve fitting techniques.

**Results:**

This study comprised 726 participants with DM who were anemic and 3,709 who were non-anemic; notably, anemic participants exhibited elevated DII scores (2.05 ± 1.50 vs. 1.70 ± 1.59). In the fully adjusted model, we noted a positive correlation between the DII and anemia (Odds Ratio [OR] = 1.09, 95% Confidence Interval [CI] =1.03 to 1.16, *p* = 0.004). Participants in the fully adjusted model had a 9% higher prevalence of anemia for each unit increase in DII. The significant association between anemia and DII persisted even after subgroup analysis. Smooth curve fitting analysis revealed a positive correlation between DII and anemia.

**Conclusion:**

There is a positive correlation between DII and anemia in individuals with DM in the United States. It provides important insights into dietary management strategies for diabetic patients with anemia.

## 1 Introduction

Anemia, as defined by the World Health Organization (WHO), occurs when there is an insufficient quantity of red blood cells (and their oxygen-carrying capacity) to meet the body’s physiological requirements, affecting about one-quarter of the world’s population and presenting a significant public health challenge (1). Anemia is prevalent among the elderly, contributing to elevated mortality and hospitalization rates in affected individuals (2). Additionally, it impacts the cognitive and behavioral development of preschool children (3, 4), acts as an independent risk factor for adverse outcomes in patients with advanced heart failure (5), and serves as an independent prognostic indicator for survival in cancer patients (6). Furthermore, anemia is a frequent but often overlooked complication of diabetes mellitus (DM), which can negatively impact the prognosis of individuals with this disease. Diabetic nephropathy, primarily leads to anemia by reducing erythropoietin (EPO) secretion (7). However, studies have shown that anemia may not only arise from diabetic kidney damage but also independently contribute to the progression of diabetic nephropathy (8, 9). The prevalence of anemia in people with DM exceeds that of the general population, highlighting its significance in influencing the quality of life and outcomes in this population (10).

Inflammatory anemia ranks among the leading causes of anemia and is particularly prevalent in patients with chronic diseases (11, 12). Cytokines induced by inflammation can impede red blood cell production, shorten their lifespan, and hinder their differentiation (13). It is important to note that maintaining adequate nutritional status is critical for supporting optimal immune system function in the body. Dietary components can directly impact inflammation levels; for instance, a nutritious diet correlates with reduced inflammatory mediator levels (14), while an unhealthy diet contributes to the onset and progression of DM. Furthermore, as research on the immune system progresses, there is growing recognition of the role of inflammation in DM development. Thus, assessing the overall inflammatory potential of individual diets can offer valuable insights into the dietary habits of people with DM.

DII (15) is a new method for evaluating dietary inflammatory potential, which can assist in assessing and shaping individual dietary habits to mitigate inflammation levels and potentially decrease the risk of specific chronic ailments. DII was first introduced by Cavicchia et al. (16) in 2009 and subsequently updated by Shivappa et al. (15) in 2014. The revised version established a global database of dietary intake across 11 representative countries spanning four continents. DII assesses dietary impacts on inflammation by correlating component intake with common markers. It examines the impact of 45 dietary components (foods, nutrients, and bioactive compounds) on inflammation markers including TNF-*α*, CRP, IL-1β, IL-4, IL-6, and IL-10. Elevated DII scores suggest a pro-inflammatory dietary pattern, indicating higher consumption of pro-inflammatory foods, while lower scores imply an anti-inflammatory dietary pattern, indicating greater consumption of anti-inflammatory foods. Numerous studies have demonstrated links between DII and the occurrence of cardiovascular diseases (17–19), overall mortality, and cancer-related mortality (20).

Moreover, recent studies indicate that inflammation is involved in a wide range of conditions that can lead to anemia (13). A prospective study indicates that gestational diabetes increases the risk of anemia in pregnant women, whereas anti-inflammatory diets may alleviate this effect, DII is significantly correlated with hemoglobin levels during the later stages of pregnancy (21). Furthermore, a study indicates that the DII is linked to anemia in U.S. adults (22); however, systematic studies on the relationship between the DII and anemia in the DM population are scarce. DM patients present unique metabolic features and a chronic low-grade inflammatory state, with dietary patterns markedly different from those of the general population. These dietary patterns may interact complexly with blood glucose control, inflammatory responses, and anemia incidence. Anemia in DM patients may accelerate the progression of related complications, particularly cardiovascular disease and diabetic nephropathy. Hence, assessing dietary inflammatory potential using DII and exploring its relationship with concurrent anemia in people with DM is vital for preventing and managing DM-related health issues. Previous studies have not thoroughly investigated the relationship between DII and anemia in individuals with DM. Our study aims to examine this correlation using publicly accessible data from the NHANES.

## 2 Materials and methods

### 2.1 Study population and design

**Figure 1:**
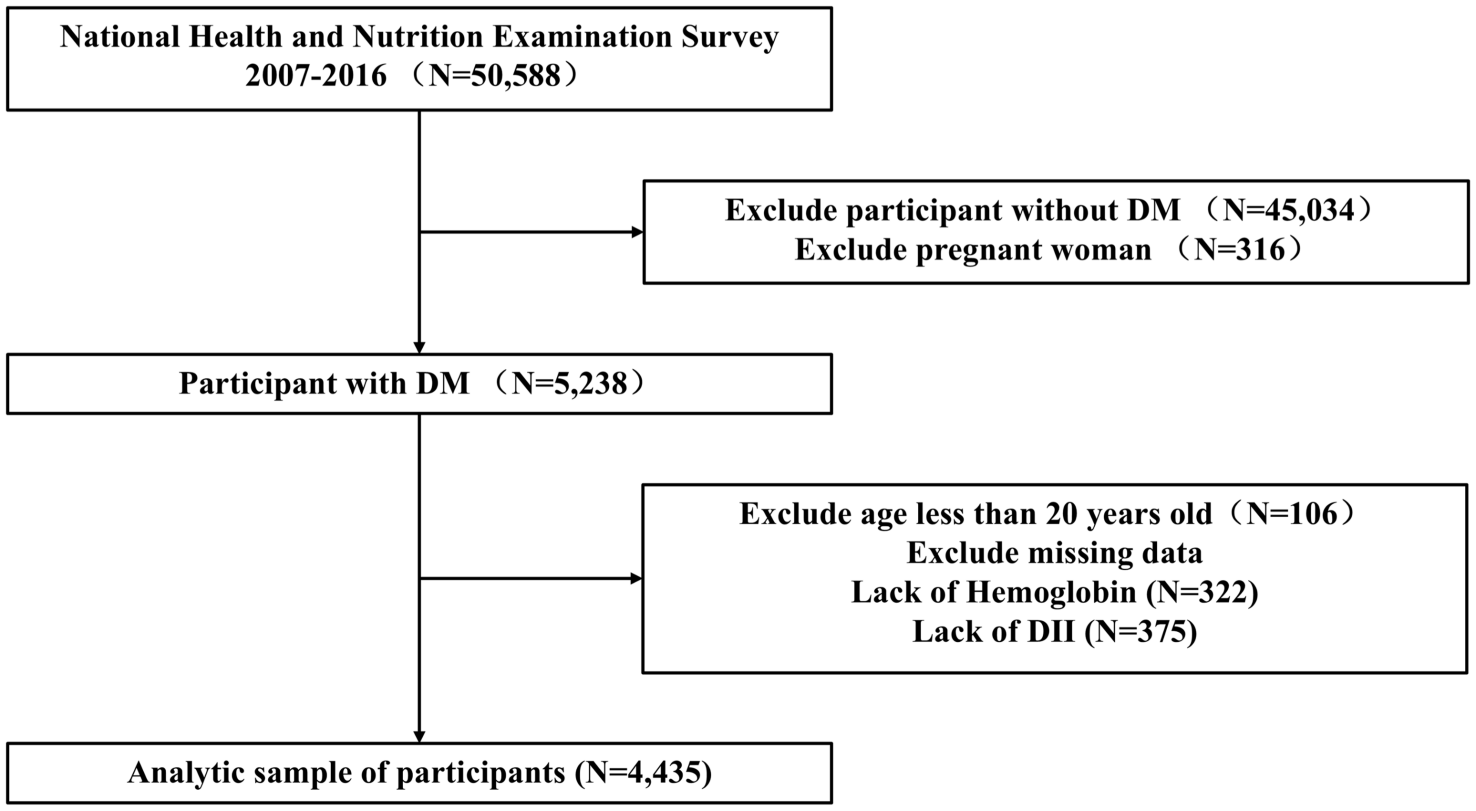
Flowchart for inclusion of participants in this study. DII, dietary inflammatory index; DM, diabetes mellitus.

The NHANES is a cross-sectional survey database that gathers data on the health and nutrition of the U.S. population. It includes multicenter data collected across the United States, updated biennially, and ensures sample representativeness through multi-stage complex sampling. This study enrolled adults aged ≥20 with DM, excluding gestational diabetes and those without hemoglobin and DII data, resulting in a final sample of 4,435 participants (Figure 1). This study received approval from the Institutional Review Board of the National Center for Health Statistics. Written informed consent was obtained from all participants. This study adheres to the Declaration of Helsinki, and our secondary analyses were conducted in accordance with the STROBE guidelines for cross-sectional studies, obviating the need for further approval from the institutional review board. For comprehensive details on NHANES methods and procedures, please visit http://www.cdc.gov/nchs/nhanes.htm.

### 2.2 Assessment of anemia

The definition of anemia, as per the WHO standards, is characterized by hemoglobin levels below 12 g/dL for females and below 13 g/dL for males (23).

### 2.3 Assessment of DM

Participants are diagnosed with DM if they meet any of the following criteria: fasting plasma glucose (FPG) ≥126 mg/dL after an 8–24-h fast; 2-h serum glucose ≥200 mg/dL following a 75 g glucose load in an oral glucose tolerance test (OGTT); HbA1c >6.5%; self-reported DM or the use of antihyperglycemic medications, as indicated in the NHANES questionnaire (24).

### 2.4 Dietary inflammatory index

The Dietary Inflammatory Index is the most widely used and authoritative tool for evaluating dietary inflammatory potential. Its structure and calculation methods have been extensively documented. The dietary parameters assessed in this study comprised 28 factors, including carbohydrates, total fat, protein, alcohol, saturated fat, fiber, cholesterol, polyunsaturated fatty acids (PUFA), monounsaturated fatty acids (MUFA), n-6 and n-3 fatty acids, thiamin, riboflavin, niacin, vitamins (A, B6, B12, C, D, and E), iron, magnesium, zinc, folic acid, selenium, caffeine, *β*-carotene, and energy content. Refer to Supplementary Table S1 for the food parameter-specific overall inflammatory effect score. Patient dietary intake information was collected through 24-h dietary recall interviews. DII was calculated based on z-scores for each dietary parameter, obtained using the formula: (average daily intake − global average daily intake)/standard deviation. These z-scores were then converted into percentiles, doubled, and subtracted from 1 to ensure a symmetrical distribution centered on 0. The final DII score was obtained by multiplying the total inflammatory impact of each dietary component and summing the results to determine the individual DII score. Participants in this study were categorized into quartiles according to their DII scores.

### 2.5 Covariates

The covariates examined in this study included: age, gender, race (Mexican American, other Hispanic, Non-Hispanic White, Other race, Non-Hispanic Black), educational attainment (less than high school, high school or equivalent, college or above), marital status (married, separated, unmarried), ratio of family income to poverty (PIR) (<1.3, 1.3–3.5, ≥3.5), body mass index (BMI) (<25, 25 ≤ BMI < 30, ≥30 kg/m^2^), smoking status, alcohol consumption, urine albumin levels, Alanine aminotransferase (ALT), Aspartate aminotransferase (AST), Serum folic acid, Serum Iron, estimated glomerular filtration rate (eGFR), hypertension, white blood cell count, Energy (kcal) and platelet count. Alcohol consumption was determined by the question: “In the past year, have you consumed at least 12 drinks of any kind of alcoholic beverage?” and smoking status was assessed by the question: “Have you smoked at least 100 cigarettes in your lifetime?” Hypertension was defined as the presence of a diagnosis of hypertension, the use of antihypertensive medications, or a mean systolic blood pressure greater than 140 mmHg and/or a mean diastolic blood pressure greater than 90 mmHg. The eGFR was determined by the CKD Epidemiology Collaboration equation (25).

### 2.6 Statistical analysis

Data analysis was conducted using R Statistics (version 4.1.3) and Empower Stats (version 4.1). The basic characteristics of the study population were described and statistically summarized by grouping according to anemia and DII. Continuous variables were presented as mean ± standard deviation (SD), while categorical variables were represented by frequency or percentage. The weighted multiple logistic regression analysis was performed to calculate Odds Ratio and 95% confidence intervals for the relationship between DII and anemia. Three models were constructed for weighted multivariate testing: Model 1 did not include any adjusted variables; Model 2 was adjusted for gender, age, and race; Model 3 was adjusted for all covariates. Smooth curve fitting and subgroup analysis were conducted to investigate the relationship between DII and anemia. Statistical significance was determined using a significance level of *p* < 0.05 for all analyses. In this study, wtdrd1/wtdrd2 was used as the final weight to adjust for variations in dietary intake data due to different times and days, ensuring accurate and reliable analysis.

## 3 Results

### 3.1 Baseline characteristics

This study enrolled 4,435 adult individuals with DM based on predetermined inclusion and exclusion criteria. The mean age of the participants was 61.64 ± 13.45 years. Of these participants, 54.84% were male, 45.16% were female, 18.17% were Mexican American, 24.62% were non-Hispanic Black, and 57.21% belonged to other races. The mean (±SD) DII value was 1.76 ± 1.58. Anemia was present in 16.37% of these participants.

Table 1 presents the clinical characteristics of all participants stratified by the presence of anemia. Individuals with DM and concurrent anemia, compared to those without anemia, exhibited higher DII scores (2.05 ± 1.50 vs. 1.70 ± 1.59), were predominantly older. Individuals in the anemia group exhibited higher platelet counts and a higher prevalence of hypertension. Additionally, anemic participants had lower serum iron, ALT, and AST levels, whereas their serum folate levels were marginally higher than those of the non-anemic group. A higher proportion of these participants had an eGFR≥60 (ml/min/1.73 m^2^). There were differences in racial distribution between the two groups, with a higher proportion of non-Hispanic Black individuals in the anemia group. Moreover, disparities were observed in education level, smoking, and alcohol consumption between the two groups. Participants with anemia had lower levels of education than those in the non-anemic group. Additionally, they showed a lower prevalence of alcohol consumption and smoking.

**Table 1:** Weighted characteristics of the study population based on anemia.

Characteristics	Overall (*n* = 4,435)	Anemia (*n* = 726)	Non-anemia (*n* = 3,709)	*p*-value
Age	61.64 ± 13.45	66.43 ± 12.50	60.70 ± 13.43	<0.001
Gender (%)				0.027
Male	2,432 (54.84%)	371 (51.10%)	2061 (55.57%)	
Female	2003 (45.16%)	355 (48.90%)	1,648 (44.43%)	
Race (%)				<0.001
Mexican American	806 (18.17%)	112 (15.43%)	694 (18.71%)	
Other Hispanic	504 (11.36%)	69 (9.50%)	435 (11.73%)	
Non-Hispanic White	1,655 (37.32%)	215 (29.61%)	1,440 (38.82%)	
Non-Hispanic Black	1,092 (24.62%)	272 (37.47%)	820 (22.11%)	
Other Race	378 (8.52%)	58 (7.99%)	320 (8.63%)	
Education level (%)				0.043
Less than high school	1,584 (35.72%)	277 (38.15%)	1,307 (35.24%)	
High School or Equivalent	1,022 (23.04%)	180 (24.79%)	842 (22.70%)	
College or above	1829 (41.24%)	269 (37.05%)	1,560 (42.06%)	
Marital status (%)				0.048
Married	3,689 (83.18%)	624 (85.95%)	3,065 (82.64%)	
Separated	156 (3.52%)	26 (3.58%)	130 (3.50%)	
Never married	590 (13.30%)	76 (10.47%)	514 (13.86%)	
PIR (%)				0.097
<1.3	1,477 (33.30%)	253 (34.85%)	1,224 (33.00%)	
1.3–3.5	2013 (45.39%)	340 (46.83%)	1,673 (45.11%)	
≥3.5	945 (21.31%)	133 (18.32%)	812 (21.89%)	
BMI (kg/m^2^) (%)				<0.001
<25	596 (13.44%)	131 (18.04%)	465 (12.54%)	
25 ≤ BMI < 30	1,284 (28.95%)	180 (24.79%)	1,104 (29.77%)	
≥30	2,555 (57.61%)	415 (57.16%)	2,140 (57.70%)	
Smoking status (%)				0.002
No	2,183 (49.22%)	395 (54.41%)	1788 (48.25%)	
Yes	2,249 (50.71%)	331 (45.59%)	1918 (51.75%)	
Drinking status (%)				<0.001
No	1,485 (33.48%)	288 (39.67%)	1,197 (32.27%)	
Yes	2,950 (66.52%)	438 (60.33%)	2,512 (67.73%)	
White blood cell (10^3^cells/uL)	7.55 ± 2.27	7.34 ± 2.30	7.59 ± 2.27	0.006
Platelet (10^3^ cells/uL)	237.51 ± 70.86	247.62 ± 85.23	235.53 ± 67.53	<0.001
Serum Iron (ug/dl)	79.37 ± 31.41	61.82 ± 30.52	82.80 ± 30.42	<0.001
AST U/L	27.42 ± 21.45	25.90 ± 32.54	27.72 ± 18.51	0.036
ALT U/L	27.60 ± 30.16	21.65 ± 16.18	28.76 ± 32.06	<0.001
Albumin (g/dl)	4.16 ± 0.34	3.97 ± 0.38	4.19 ± 0.32	<0.001
eGFR (ml/min/1.73 m^2^) (%)				<0.001
<60	889 (20.05%)	301 (41.46%)	588 (15.85%)	
≥60	3,546 (79.95%)	425 (58.54%)	3,121 (84.15%)	
Folic acid (ng/ml)	21.58 ± 15.37	22.64 ± 17.82	21.38 ± 14.83	0.043
DII	1.76 ± 1.58	2.05 ± 1.50	1.70 ± 1.59	<0.001
Hypertension (%)				<0.001
No	1,214 (27.37%)	119 (16.39%)	1,095 (29.52%)	
Yes	3,221 (72.63%)	607 (83.61%)	2,614 (70.48%)	
Energy (kcal)	1877.55 ± 889.55	1686.69 ± 776.06	1914.91 ± 905.51	<0.001

Mean ± SD for continuous variables; % for categorical variables. BMI, body mass index; DII, dietary inflammatory index; PIR, ratio of family income to poverty; ALT, alanine aminotransferase; AST, aspartate aminotransferase; eGFR, estimated glomerular filtration rate.

Table 2 presents the clinical characteristics of all participants stratified by DII quartiles. The DII quartiles ranged from <0.750, 0.750 to 1.966, 1.966 to 2.974, and ≥ 2.974. The proportion of anemic individuals increased with higher DII quartiles (Quartile 1: 12.80%, Quartile 2: 14.88%, Quartile 3: 17.06%, Quartile 4: 20.74%; *p* < 0.001). Moreover, disparities were observed in gender, race, education level, PIR, BMI, alcohol consumption, white blood cell count, energy and hypertension across different DII quartiles. The proportion of female patients and white blood cell count increased with higher DII quartiles. As the DII index increased, energy intake and albumin levels among participants gradually declined. Furthermore, among participants with eGFR<60 (ml/min/1.73 m^2^), the proportion increased as their DII rose.

**Table 2:** Weighted characteristics of the study population based on DII quartiles.

Characteristics	DII quartiles				*p*-value
	Quartile 1	Quartile 2	Quartile 3	Quartile 4	
	<0.750	0.750–1.966	1.966–2.974	≥2.974	
Age	61.17 ± 13.23	61.27 ± 13.52	61.18 ± 13.55	62.94 ± 13.44	0.003
Gender (%)					<0.001
Male	764 (68.89%)	663 (59.78%)	543 (49.01%)	462 (41.66%)	
Female	345 (31.11%)	446 (40.22%)	565 (50.99%)	647 (58.34%)	
Race (%)					<0.001
Mexican American	217 (19.57%)	209 (18.85%)	198 (17.87%)	182 (16.41%)	
Other Hispanic	105 (9.47%)	119 (10.73%)	155 (13.99%)	125 (11.27%)	
Non-Hispanic White	447 (40.31%)	436 (39.31%)	372 (33.57%)	400 (36.07%)	
Non-Hispanic Black	210 (18.94%)	260 (23.44%)	287 (25.90%)	335 (30.21%)	
Other Race	130 (11.72%)	85 (7.66%)	96 (8.66%)	67 (6.04%)	
Education level (%)					<0.001
Less than high school	283 (25.52%)	360 (32.46%)	415 (37.45%)	526 (47.43%)	
High School or Equivalent	243 (21.91%)	286 (25.79%)	247 (22.29%)	246 (22.18%)	
College or above	583 (52.57%)	463 (41.75%)	446 (40.25%)	337 (30.39%)	
Marital status (%)					0.234
Married	942 (84.94%)	928 (83.68%)	910 (82.13%)	909 (81.97%)	
Separated	28 (2.52%)	43 (3.88%)	46 (4.15%)	39 (3.52%)	
Never married	139 (12.53%)	138 (12.44%)	152 (13.72%)	161 (14.52%)	
PIR (%)					<0.001
<1.3	265 (23.90%)	349 (31.47%)	395 (35.65%)	468 (42.20%)	
1.3–3.5	507 (45.72%)	506 (45.63%)	508 (45.85%)	492 (44.36%)	
≥3.5	337 (30.39%)	254 (22.90%)	205 (18.50%)	149 (13.44%)	
BMI (kg/m^2^) (%)					0.029
<25	167 (15.06%)	131 (11.81%)	153 (13.81%)	145 (13.07%)	
25 ≤ BMI < 30	336 (30.30%)	347 (31.29%)	289 (26.08%)	312 (28.13%)	
≥30	606 (54.64%)	631 (56.90%)	666 (60.11%)	652 (58.79%)	
Smoking status (%)					0.101
No	581 (52.39%)	527 (47.52%)	539 (48.65%)	536 (48.33%)	
Yes	528 (47.61%)	582 (52.48%)	569 (51.35%)	573 (51.67%)	
Drinking status (%)					<0.001
No	300 (27.05%)	362 (32.64%)	379 (34.21%)	444 (40.04%)	
Yes	809 (72.95%)	747 (67.36%)	729 (65.79%)	665 (59.96%)	
White blood cell (10^3^cells/uL)	7.32 ± 1.94	7.53 ± 2.36	7.65 ± 2.34	7.69 ± 2.41	<0.001
Platelet (10^3^ cells/uL)	232.43 ± 69.06	233.91 ± 69.18	242.06 ± 72.61	241.63 ± 72.07	<0.001
Serum Iron (ug/dl)	80.78 ± 30.57	80.76 ± 31.46	78.94 ± 30.27	77.00 ± 33.15	0.012
AST U/L	27.80 ± 17.75	27.70 ± 15.50	27.29 ± 22.40	26.90 ± 27.99	0.746
ALT U/L	29.50 ± 30.76	27.70 ± 16.69	28.50 ± 45.47	24.68 ± 18.32	0.001
Albumin (g/dl)	4.20 ± 0.35	4.19 ± 0.32	4.14 ± 0.35	4.10 ± 0.33	<0.001
eGFR (ml/min/1.73 m^2^)					<0.001
<60	172 (15.51%)	200 (18.03%)	239 (21.57%)	278 (25.07%)	
≥60	937 (84.49%)	909 (81.97%)	869 (78.43%)	831 (74.93%)	
Folic acid (ng/ml)	22.96 ± 17.47	20.91 ± 8.93	21.54 ± 18.96	20.92 ± 14.07	0.004
Anemia (%)					<0.001
No	967 (87.20%)	944 (85.12%)	919 (82.94%)	879 (79.26%)	
Yes	142 (12.80%)	165 (14.88%)	189 (17.06%)	230 (20.74%)	
Hypertension (%)					0.005
No	329 (29.67%)	329 (29.67%)	288 (25.99%)	268 (24.17%)	
Yes	780 (70.33%)	780 (70.33%)	820 (74.01%)	841 (75.83%)	
Energy (kcal)	2467.05 ± 1008.65	2049.78 ± 786.62	1713.66 ± 672.46	1279.56 ± 562.52	<0.001

Mean ± SD for continuous variables; % for categorical variables. BMI, body mass index; DII, dietary inflammatory index; PIR, ratio of family income to poverty; ALT, Alanine aminotransferase; AST, Aspartate aminotransferase; eGFR, estimated glomerular filtration rate.

### 3.2 Associations between DII and anemia

**Figure 2:**
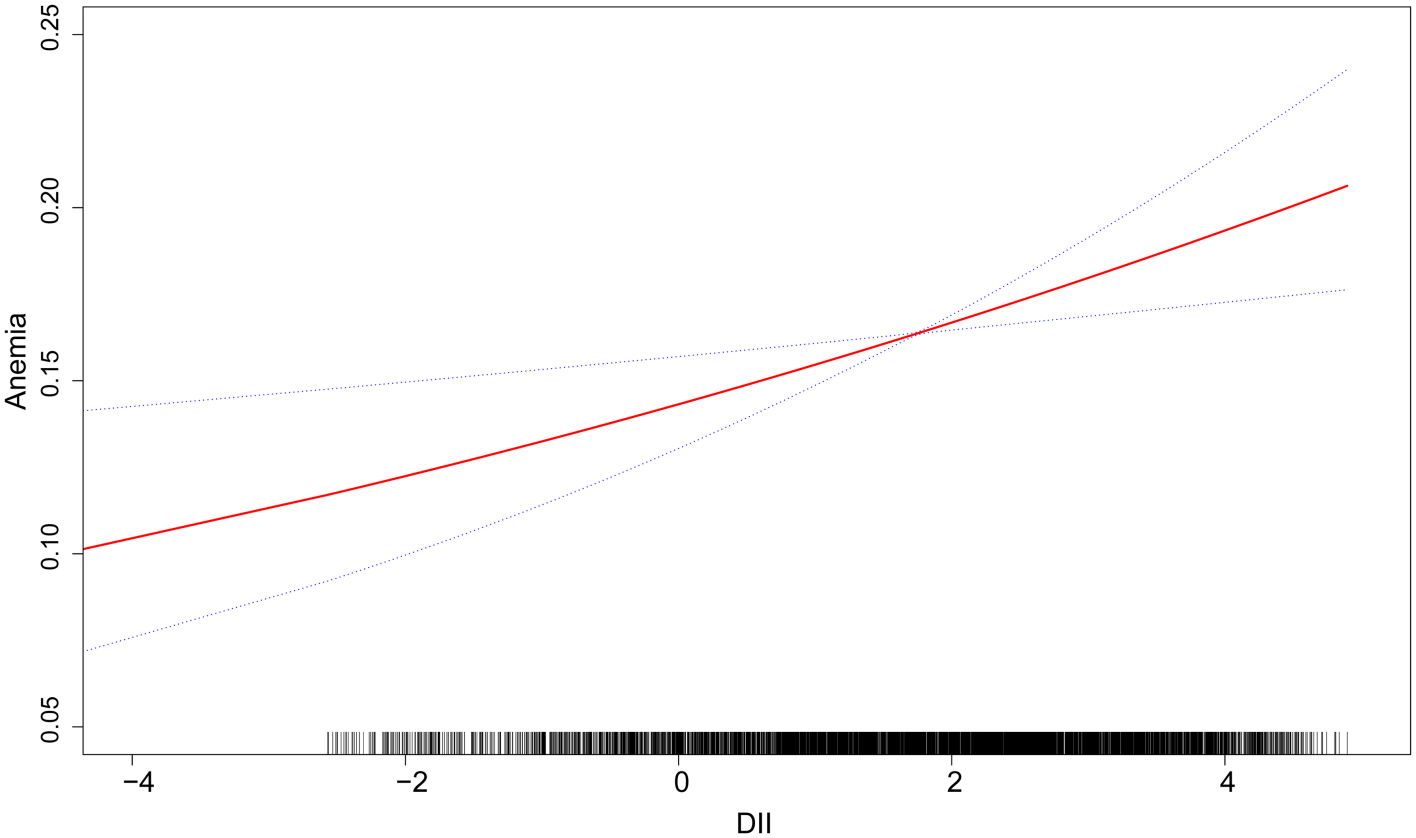
The linear associations between DII and anemia. The solid red line illustrates the smooth curve fitting. Blue bands indicate the 95% confidence interval of the fit.

Table 3 displays the results of multivariate weighted logistic regression. Both unadjusted and adjusted models show a significant positive correlation between DII and anemia: Model 1 [1.16 (1.10, 1.22) <0.001], Model 2 [1.12 (1.06, 1.18) <0.001], Model 3 [1.09 (1.03, 1.16) 0.004]. Furthermore, a higher prevalence of anemia was observed in the fourth quartile in Model 1 [1.78 (1.12, 2.24), *p* < 0.001], Model 2 [1.53 (1.21, 1.94), *p* < 0.001], and Model 3 [1.45 (1.11, 1.90) 0.007]. The findings indicate that in the fully adjusted model, participants had a 9% higher prevalence of anemia for each unit increase in DII. Additionally, following adjustments for gender, age, race, education level, marital status, smoking habits, alcohol consumption habits, albumin, hypertension, white blood cells, platelets, PIR, BMI, ALT, AST, Serum folic acid, Serum Iron, Energy and eGFR, the smoothed curve fitting analysis reveals a notable positive correlation between DII and anemia (Figure 2).

**Table 3:** The odds ratio for the relationship between DII and anemia.

Exposure	*OR* (95%CI), *p*-value		
	Model 1	Model 2	Model 3
DII	1.16 (1.10,1.22) <0.001	1.12 (1.06, 1.18) <0.001	1.09 (1.03, 1.16) 0.004
DII Quintiles			
Quartile 1	Reference	Reference	Reference
Quartile 2	1.19 (0.93, 1.52) 0.158	1.15 (0.89, 1.47) 0.285	1.25 (0.95, 1.76) 0.110
Quartile 3	1.40 (1.15, 1.81) 0.005	1.30 (1.02, 1.66) 0.035	1.30 (0.99, 1.71) 0.056
Quartile 4	1.78 (1.12, 2.24) <0.001	1.53 (1.21, 1.94) <0.001	1.45 (1.11, 1.90) 0.007
*p* for trend	<0.001	<0.001	0.009

Model 1: No covariates were adjusted. Model 2: Age, Gender, Race were adjusted. Model 3: Age, Gender, Race, Education level, Marital status, PIR, Drinking, Smoking, BMI, White blood cell, Platelet, Albumin, Serum Iron, AST, ALT, Folic acid, eGFR, Hypertension, Energy were adjusted. 95% CI, 95% confidence interval; OR, odds ratio; DII, dietary inflammatory index.

### 3.3 Subgroup analysis

**Figure 3:**
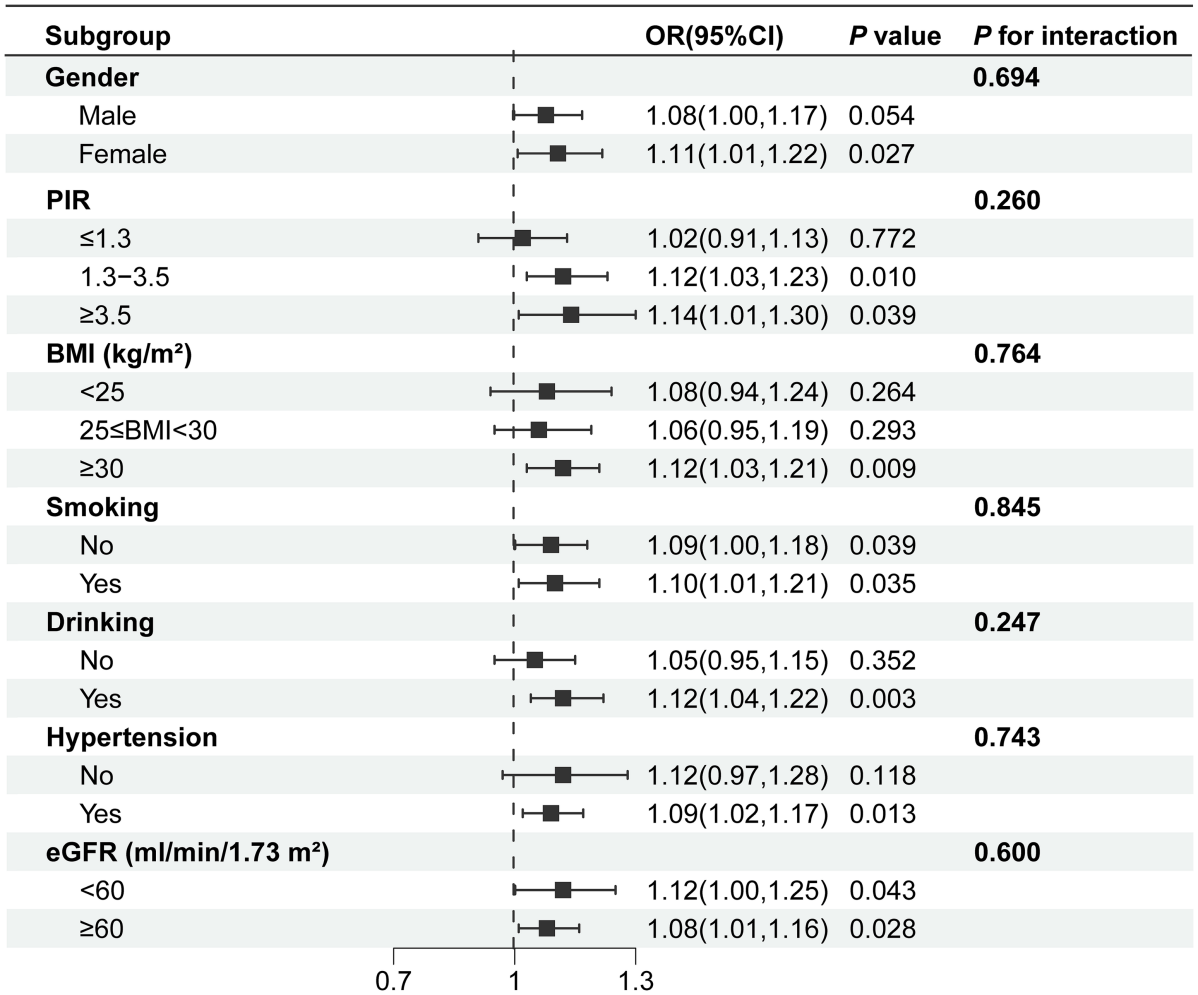
Subgroup analysis of the association between DII and anemia. Age, gender, race, education level, marital status, PIR, drinking, smoking, BMI, white blood cell, platelet, albumin, hypertension, ALT, AST, serum folic acid, energy, serum iron, eGFR were adjusted. BMI, body mass index; PIR, ratio of family income to poverty; eGFR, estimated glomerular filtration rate.

Subgroup analysis was conducted based on gender, PIR, BMI, smoking status, alcohol consumption status, eGFR and hypertension to delve deeper into the association between DII and anemia, as illustrated in Figure 3. The findings revealed a positive correlation between DII and anemia in different groups. In the gender subgroups, each unit increase in the DII was associated with an 11% increase in anemia prevalence among female participants, whereas no such association was found in male participants. Concerning household income, a correlation between DII and anemia was noted when PIR was greater than 1.3. When the PIR was greater than 3.5, the prevalence of anemia increased by 14% for each unit increase in DII. In BMI subgroups, a correlation between DII and anemia was observed for BMI ≥ 30, while no correlation was found for BMI < 25 and 25 ≤ BMI < 30. In eGFR subgroups, both DII and anemia demonstrate a significant positive correlation: eGFR≥60 (ml/min/1.73 m^2^) [1.08 (1.01, 1.16), *p* < 0.05], eGFR<60 (ml/min/1.73 m^2^) [1.12 (1.00, 1.25), *p* < 0.05]. Furthermore, the study revealed that among individuals with diabetes who were drinkers, and had hypertension, the correlation between DII and anemia was more pronounced.

## 4 Discussion

In this cross-sectional study involving 4,435 individuals, researchers discovered a positive correlation between the DII and anemia among individuals with DM, suggesting that higher DII values are associated with an increased prevalence of anemia. Hence, optimizing the dietary composition of individuals with DM to mitigate the inflammatory potential of their diet might serve as an adjunctive approach to prevent anemia as a complication of DM.

In recent times, there has been growing attention towards the relationship between dietary patterns and chronic diseases (26). DII has emerged as a novel analytical tool for assessing the overall inflammatory potential of an individual’s diet. It facilitates the evaluation of the relationship between dietary patterns, inflammatory factors, and health outcomes in chronic diseases. A HELENA study revealed that diets high in sugars and saturated fats are positively correlated with elevated levels of inflammatory factors such as TNF-*α*, IL-2, and INF-*γ*. Conversely, increased consumption of grains and vegetables is associated with lower CRP levels (27). Additionally, data from the European Perspective Investigation into Cancer and Nutrition (EPIC) cohort study showed a positive correlation between the DII and CRP, IL-6, and TNF-α levels, supporting its use in assessing the inflammatory potential of diets among European adults (28). These findings emphasize the significance of dietary interventions in managing systemic inflammation.

A study has identified an inflammatory dietary pattern associated with a high DII, characterized by high consumption of red meat, saturated fats, and processed foods, coupled with low intake of anti-inflammatory fruits and vegetables. The study found that a high intake of inflammatory foods is associated with an increased risk of anemia (29). Ethnic groups exhibit distinct dietary patterns. In the United States, Black individuals consume significantly fewer fruits and vegetables than other ethnic groups (30). But studies have shown that the incidence of inflammatory anemia is notably higher among Black Americans than White Americans (31). Furthermore, pregnant women often consume iron-rich foods and supplements, including red meat, to prevent anemia. However, red meat has pro-inflammatory effects. However, pregnant women with gestational diabetes and elevated inflammatory markers are more likely to develop anemia despite this intervention. Researchers suggest that a low-DII, anti-inflammatory dietary pattern may counteract inflammation induced by gestational diabetes mellitus (GDM), potentially reducing heparin release and the incidence of anemia (28). Therefore, a low-DII, anti-inflammatory dietary pattern may help reduce factors contributing to anemia.

Individuals with DM often develop early-stage renal impairment, which is a major cause of renal anemia (32). Additionally, individuals with DM have a higher prevalence of anemia compared to those without DM (33). Despite numerous studies demonstrating a higher prevalence of anemia in individuals with DM, these studies primarily included populations with renal impairment (34). Merlin C and colleagues conducted a cross-sectional study involving 820 patients with DM, including those with normal albuminuria and glomerular filtration rate (GFR) as well as those with overt nephropathy, thereby enhancing the representativeness of the study population. The study found that 23% of patients had undiagnosed anemia, which was two to three times more common than in the general population of patients with similar levels of renal impairment and iron stores (35). Therefore, other factors related to DM contribute to the increased risk of anemia in individuals with DM. Daniel et al. also reported that anemia unrelated to renal impairment is common in patients with DM and prediabetes (36). Consequently, individuals with DM have higher rates and greater severity of anemia compared to those without DM, regardless of renal impairment, highlighting the multifaceted etiology of anemia in DM (37).

Anemia in DM is multifactorial, involving renal dysfunction, EPO deficiency, chronic inflammation, and insulin resistance in type 2 diabetes, while autoimmune diseases are significant factors in type 1 diabetes (38–42). Notably, inflammatory anemia is a common cause of anemia subsequent to iron deficiency anemia. Inflammation induces hepcidin, which suppresses the expression of iron transport proteins, leading to iron sequestration in macrophages and subsequent erythropoietic iron restriction (43). Furthermore, hepcidin is inversely correlated with duodenal iron transporter expression, affecting iron absorption (44). Additionally, elevated levels of inflammatory cytokines, such as interleukin-6 (IL-6) and C-reactive protein, in patients with inflammatory anemia may hinder the production and function of EPO, thus impeding normal proliferation and differentiation of erythroid progenitor cells (1, 45). Cytokines degrade senescent red blood cells, and inflammation-induced free radicals further shorten the half-life of red blood cells (46). In this regard, the dietary habits of individuals with DM can significantly affect the chronic inflammatory state of the body. Ikuyo et al. found a correlation between elevated DII scores and reduced late-pregnancy hemoglobin levels in pregnant women, suggesting that anti-inflammatory diets might alleviate inflammation caused by GDM and decrease the prevalence of anemia (21).

Therefore, regulating pro-inflammatory diet intake may be critical for preventing anemia in diabetic patients. Several prospective studies have found an inverse association between adherence to the anti-inflammatory Mediterranean diet and the risk of DM (47). Studies on dietary patterns and inflammation have shown that red meat, processed meats, refined grains, and sugary beverages exert pro-inflammatory effects, whereas whole grains, fruits, and vegetables are anti-inflammatory (48). However, although foods like grains, fruits, and vegetables have lower DII scores, strict vegetarians are more susceptible to iron-deficiency anemia. A balanced and healthy diet should be promoted, with appropriate intake of refined carbohydrates, reduced total fatty acids, increased *ω*-3 fatty acids and fiber, and higher consumption of antioxidant vitamins (49).

This study has several strengths. Firstly, it is based on the NHANES database, which offers a large, nationally representative sample. Sample weights were incorporated into the analysis to ensure representativeness. Furthermore, the study adjusted for potential confounding variables and conducted subgroup analyses to assess their impact on the results. However, there are also limitations. This study is cross-sectional and cannot establish causal relationships between study variables or predict disease risks. Although some potential confounding variables were adjusted for, the authors could not rule out the influence of other possible confounders. Finally, we were unable to analyze the correlation between the DII and specific types of anemia, such as renal anemia and iron-deficiency anemia, due to the limited sample size of laboratory test indicators in the database. We intend to conduct a future study to recruit a larger number of participants diagnosed with DM, while also gathering additional indicators (serum vitamin B12, iron metabolism, EPO, etc.) to further validate our findings.

## 5 Conclusion

This study uncovered a positive correlation between the DII and anemia among individuals diagnosed with DM. Higher DII scores are associated with higher prevalence of anemia in individuals. If the causal relationship between them can be verified through large-scale clinical trials in the future, it will have significant implications for preventive care in diabetic patients. The DII could be incorporated into anemia prevention and treatment strategies, and nutritional interventions could be promoted, particularly for high-risk populations, to adopt anti-inflammatory diets as a preventive measure.

## Data Availability

The original contributions presented in the study are included in the article/Supplementary material, further inquiries can be directed to the corresponding authors.
