# Supplementary material for: The association between dietary inflammatory index and anemia in individuals with diabetes mellitus
Source: Front Nutr. 2025 Feb 17;12:1538696. doi: 10.3389/fnut.2025.1538696 (PMC11874837; doi:10.3389/fnut.2025.1538696)
Supplement: Supplementary file 1 [file Table_1.docx]

**Supplemental Table 1** Overall Inflammatory Effect Score for 28 Specific Food Parameters

| **Food** | **Overall inflammatory effect score** |
| --- | --- |
| Alcohol (g) | −0.278 |
| Vitamin B12 (μg) | 0.106 |
| Vitamin B6 (mg) | −0.365 |
| β-Carotene (μg) | −0.584 |
| Caffeine (g) | −0.110 |
| Carbohydrate (g) | 0.097 |
| Cholesterol (mg) | 0.110 |
| Energy (kcal) | 0.180 |
| Total fat (g) | 0.298 |
| Fibre (g) | −0.663 |
| Folic acid (μg) | −0.190 |
| Fe (mg) | 0.032 |
| Mg (mg) | −0.484 |
| MUFA (g) | −0.009 |
| Niacin (mg) | −0.246 |
| *n*-3 Fatty acids (g)  *n*-6 Fatty acids (g) | −0.436  −0.159 |
| Protein (g) | 0.021 |
| PUFA (g) | −0.337 |
| Riboflavin (mg) | −0.068 |
| Saturated fat (g) | 0.373 |
| Se (μg) | −0.191 |
| Thiamin (mg) | −0.098 |
| Vitamin A (RE) | −0.401 |
| Vitamin C (mg)  Vitamin D (μg) | −0.424  −0.446 |
| Vitamin E (mg) | −0.419 |
| Zn (mg) | −0.313 |
